# Novel Cationic Carotenoid Lipids as Delivery Vectors of Antisense Oligonucleotides for Exon Skipping in Duchenne Muscular Dystrophy

**DOI:** 10.3390/molecules17021138

**Published:** 2012-01-24

**Authors:** Linda J. Popplewell, Aseel Abu-Dayya, Tushar Khanna, Marcella Flinterman, Nada Abdul Khalique, Liji Raju, Christer L. Øpstad, Hans-Richard Sliwka, Vassilia Partali, George Dickson, Michael D. Pungente

**Affiliations:** 1 School of Biological Sciences, Royal Holloway-University of London, Egham, Surrey TW20 0EX, UK; 2 Research Division, Weill Cornell Medical College in Qatar, Doha, P.O. Box 24144, Qatar; 3 Department of Chemistry, Norwegian University of Science and Technology (NTNU), 7491 Trondheim, Norway; 4 Pre-Medical Unit, Weill Cornell Medical College in Qatar, Doha, P.O. Box 24144, Qatar

**Keywords:** cationic lipid, carotenoid, antisense oligonucleotide, exon skipping, Duchenne muscular dystrophy

## Abstract

Duchenne Muscular Dystrophy (DMD) is a common, inherited, incurable, fatal muscle wasting disease caused by deletions that disrupt the reading frame of the DMD gene such that no functional dystrophin protein is produced. Antisense oligonucleotide (AO)-directed exon skipping restores the reading frame of the *DMD* gene, and truncated, yet functional dystrophin protein is expressed. The aim of this study was to assess the efficiency of two novel rigid, cationic carotenoid lipids, C30-20 and C20-20, in the delivery of a phosphorodiamidate morpholino (PMO) AO, specifically designed for the targeted skipping of exon 45 of *DMD* mRNA in normal human skeletal muscle primary cells (hSkMCs). The cationic carotenoid lipid/PMO-AO lipoplexes yielded significant exon 45 skipping relative to a known commercial lipid, 1,2-dimyristoyl-*sn*-glycero-3-ethylphosphocholine (EPC).

## 1. Introduction

In a physiological context, carotenoids are best known as antioxidants electron donors to noxious radicals [1] and quenchers of harmful singlet oxygen [2]. Carotenoids show further anticancer [3] and antistroke activity [4], besides other not yet fully exploited therapies [5], together with the precedent article we present here, to the best of our knowledge, the first application of carotenoids in gene transfection.

Antisense oligonucleotides (AOs) are single-stranded DNA molecules, typically 13–25 nucleotides in length, synthesized to be complimentary to a unique RNA sequence. Antisense therapy is a form of treatment for genetic disorders for which a mutation of the genetic sequence of a particular gene is known to be responsible for a particular disease. In antisense therapy, the AO binds to the messenger RNA (mRNA) produced by that gene to inactivate it. Alternatively, the AO might be targeted to bind a splicing region on a pre-mRNA and modify the defective exon content of an mRNA so as to open the reading frame and allow the production of an essential protein product.

Interest in AO therapy has recently focused on addressing vital medical needs, such as cancer, viral infections, muscular and neurological diseases. Duchenne Muscular Dystrophy (DMD) is an incurable muscle wasting disease caused by a vast number of deletions that disrupt the reading frame of the *DMD* gene, so that no functional dystrophin protein is produced. Antisense oligonucleotide (AO) directed exon skipping restores the reading frame of the *DMD* gene, and truncated, yet functional dystrophin protein is expressed. While recent reports have shown the potential for AOs to ameliorate DMD [6,7,8,9,10,11], numerous anatomic and cellular barriers remain that prevent this therapy from realizing its full potential. Much effort over recent years has been focused on improving the delivery of AOs using both viral and non-viral carriers. While viral delivery is generally regarded as more effective, this approach has a number of drawbacks, specifically large-scale production cost, limited cargo capacity, safety concerns and the potential for an immune response against the viral vector. Although non-viral agents such as lipids and polymers have much lower transfection efficiencies compared to their viral counterparts, they are cheaper and easier to produce in bulk, they are not limited in their cargo capacity and can be administered repeatedly.

**Figure 1:**
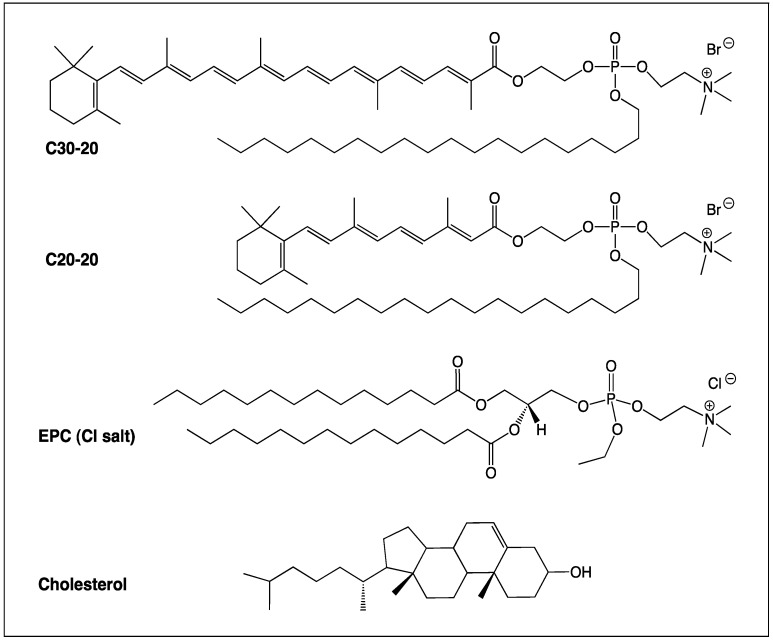
Structures of cationic carotenoid lipids C30-20 and C20-20, together with commercially available cationic lipid EPC, and neutral co-lipid, cholesterol.

In the present study we report what is, to our knowledge, the first use of carotenoid-derived cationic lipids as delivery vectors of genetic material. Two novel cationic lipids, C30-20 and C20-20 (Figure 1), synthesized from carotenoic and retinoic acid (C30- and C20-acid), respectively, combined with a flexible icosan-1-ol chain coupled through a phosphocholine headgroup, were used to formulate lipoplexes with phosphorodiamidate morpholino (PMO) AOs designed to specifically target exon 45 skipping of the *DMD* mRNA and delivered into normal human skeletal muscle primary cells (hSkMCs). Skipping of exon 45 would have the potential to treat 8.1% of DMD patients, the second highest percentage of patients after skipping of exon 51 (13%) [12].

## 2. Results and Discussion

### 2.1. Synthesis of the Cationic Carotenoid Lipids C30-20 and C20-20

The carotenoid lipids C30-20 and C20-20 were synthesized from commercial C30-carotenoid ester and C20-acid (retinoic acid). All intermediates and final products were purified after each step and fully characterized by thin-layer chromatography, ultraviolet-visible spectroscopy, low and high resolution mass spectrometry, ^1^H-, ^13^C- and ^31^P-NMR. All analytical data were found to be in accordance with known related compounds previously synthesized in our lab, and the detailed synthesis of C30-20 and C20-20 will be published elsewhere, together with the synthesis of other lipids of the C30-n and C20-n series. The structures of C30-20, C30-30, 1,2-dimyristoyl-*sn*-glycero-3-ethylphosphocholine (EPC) and cholesterol are shown in Figure 1.

### 2.2. Verification of Annealing PMO to Leash

Verification that the leash had annealed to the PMO was confirmed by agarose gel electrophoresis. Aliquots of leash alone and leashed PMO were run on a 3% agarose gel. The PMO and leash had annealed successfully as an increase in size (*i.e.*, slower moving band) was evident in leashed PMO relative to PMO alone (results not shown).

### 2.3. Gel Retardation Assay

**Figure 2:**
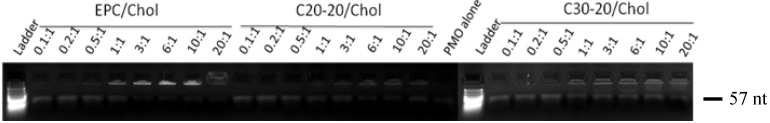
Gel retardation assay of EPC/Chol/PMO AO, C20-20/Chol/PMO AO and C30-20/Chol/PMO AO at (+/−) molar charge ratios 0.1:1 up to 20:1; low molecular weight marker run through a 3% TBE-agarose gel impregnated with the DNA gel stain, ethidium bromide. The gel was visualized using a Geliance transilluminator.

PMO are uncharged molecules and will not migrate in agarose in a manner comparative to conventional deoxynucleic acids. The leash moiety does add charge and allows electrophoretic migration, thus enabling gel retardation evaluation. However, molecular weight comparisons with dsDNA migration markers cannot be made. Nevertheless, the size designation of 57 nt is denoted beside the gel image in Figure 2, as this corresponds to the 30 mer PMO annealed with the 27 mer leash that contains 17 complimentary bases, leaving a 10 mer overhang. Complexation of negatively charged leashed PMO-AO with the cationic lipids results in a neutral particle once the negative charges of the nucleotides are paired with an equimolar amount of positively charged lipid molecules. Full gel retardation would then be expected once this equimolar pairing of (+/−) charges is achieved. However, structural and physicochemical differences between lipids may result in different packing properties of the various lipid/PMO-AO complexes. Complete gel retardation is not always observed with a 1:1 +/− charge ratio, suggesting that the properties of the various lipid/PMO AO lipoplexes are sensitive to the nature of the cationic lipid. 

Lipid/PMO AO lipoplexes were prepared at various (+/−) molar charge ratios (nitrogen/phosphorus, or N/P ratios) ranging from 20:1–0.1:1 for lipoplexes EPC/Chol/PMO, C30-20/Chol/PMO and C20-20/Chol/PMO. The results of the gel retardation assay revealed that the C20-20/Chol/PMO complex resulted in the highest level of retention at a charge ratio of 20:1 (Figure 2). Neither the EPC/Chol/PMO or C30-20/Chol/PMO lipoplexes revealed complete retention even at charge ratios as high as 20:1.

### 2.4. Qualitative Assessment of Cell Viability

**Figure 3:**
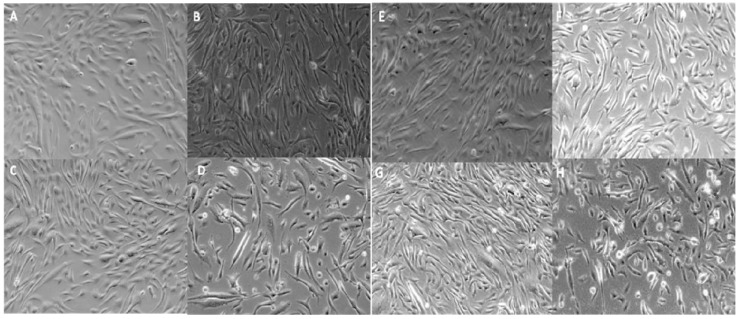
Qualitative cell viability assessment by light microscopy of hSkMCs transfected with lipoplex formulations C20-20/Chol/PMO AO (**A**–**D**) and C30-20/Chol/PMO AO (**E**–**H**) at (+/−) molar charge ratios of 0.05:1 (**A**, **E**), 0.1:1 (**B**, **F**) 0.25:1 (**C**, **G**) and 0.5:1 (**D**, **H**) at 24 h.

At low (+/−) molar charge ratios (0.05:1 up to 0.25:1), the carotenoid lipid/PMO lipoplexes were well tolerated by hSkMCs upon visual inspection at 24 h, but some cell toxicity was evident at the higher (+/−) molar charge ratio of 0.5:1 for both carotenoid lipid/PMO lipoplexes tested (Figure 3).

### 2.5. Reverse Transcriptase-PCR Results

To verify the efficiency of the lipoplex formulations for delivery of the targeted AO, hSkMCs were transfected with PMO AO oligomers specifically targeted for skipping exon 45 of the mRNA, and RNA was extracted after 24 h. As the levels of skipped transcript are generally very small relative to the full-length transcript, nested reverse transcriptase-PCR (RT-PCR) on the harvested RNA was required. RT-PCR was performed on 200 ng RNA from hSkMCs treated with three different lipoplex formulas, namely EPC/Chol/PMO, C30-20/Chol/PMO and C20-20/Chol/PMO. 

**Figure 4:**
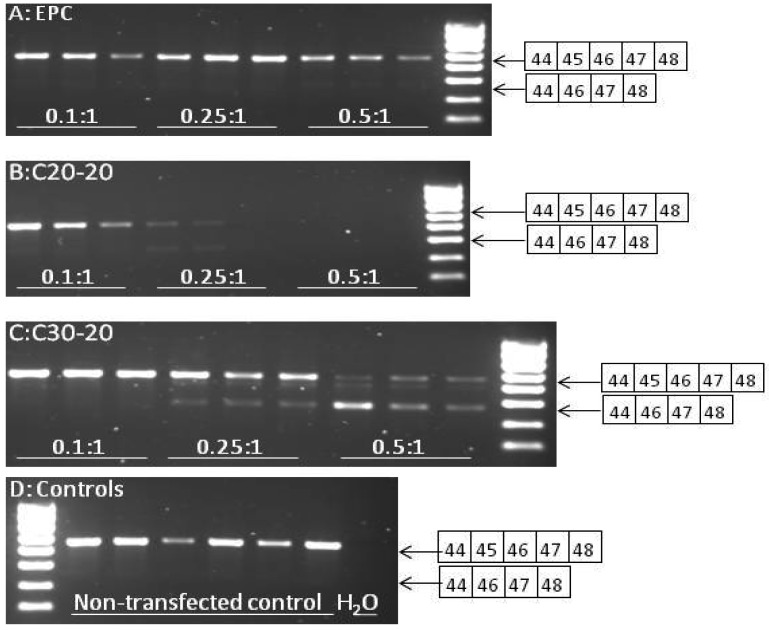
(**A**)–(**C**) Comparison of efficiency of various lipid/PMO AO complexes (each performed in triplicate) to induce skipping of exon 45 in RNA from hSkMCs. Nested RT-PCR was performed on 200 ng from hSkMCs treated with lipoplex formulations, EPC/Chol/PMO (**A**), C20-20/Chol/PMO (B) and C30-20/Chol/PMO (**C**) at 250 nmol/L at the ratios indicated. The obtained products were separated by agarose gel (1.5%) electrophoresis against Hyper ladder IV. The full-length product (exons 44-48) is 657 bp and the skipped product (exons 44, 46-48) is 481 bp in size. Non-transfected controls are shown in **D**.

Agarose gel electrophoresis separation of products for charge ratios up to 0.5:1 for each lipid is shown in Figure 4 (results not shown for charge ratios 0.05:1). 

**Figure 5:**
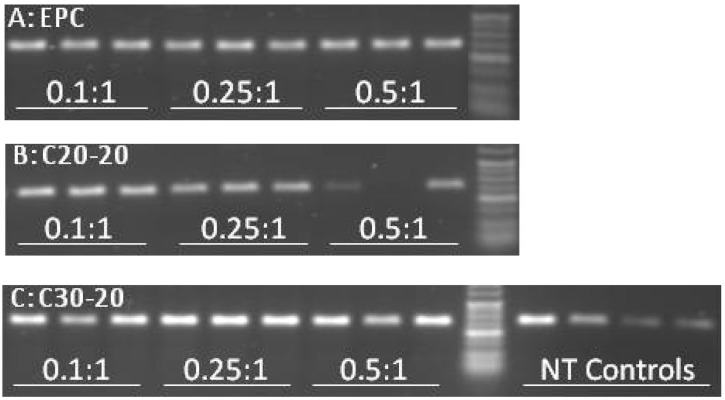
RT-PCR amplification of ribosomal 18s housekeeping gene to assess comparative RNA quality between samples transfected with various lipid/PMO AO complexes. 100 ng of RNA harvested from hSkMCs treated with lipoplex formulations, EPC/Chol/PMO, C20-20/Chol/PMO and C30-20/Chol/PMO at 250 nmol/L at the ratios indicated was subjected to RT-PCR amplification. The obtained products were separated by agarose gel (2.5%) electrophoresis against Hyper ladder V. The expected product is around 130 bp in size.

Future work will therefore include time-course studies to establish in further detail the efficacy of our novel vectors. To normalize for RNA quality within the RT-PCR assay, amplification of the housekeeping gene, ribosomal 18s was also performed (Figure 5, results not shown for charge ratios 0.05:1). 

**Figure 6:**
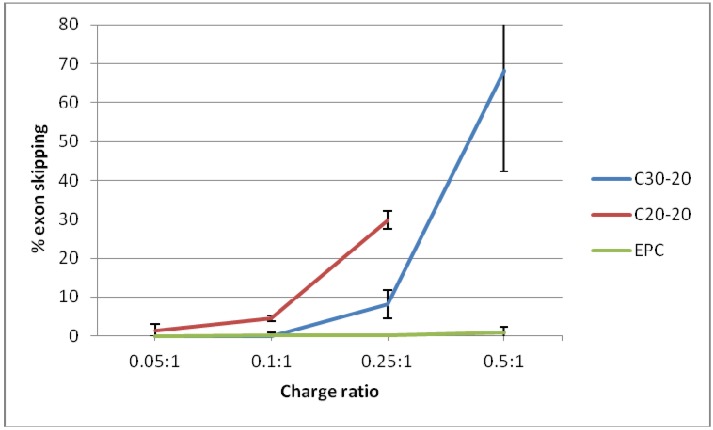
Comparison of lipoplex formulations (EPC/Chol/PMO, C30-20/Chol/PMO and C20-20/Chol/PMO at 250 nmol/L) to produce exon 45 skipping in hSkMCs over a range of charge ratios.

Equal amounts of RNA (100 ng) were subjected to RT-PCR amplification; the poor quality of RNA harvested from cells transfected with C20-20/Chol/PMO at a charge ratio of 0.5:1 is evident. This explains the failure of exon 44-48 amplification by nested RT-PCR in these samples. The poor RNA quality is likely to be the result of the toxicity seen for C20-20/Chol/PMO at the charge ratio of 0.5:1 (Figure 3, **D**). Semi-quantification of levels of skipping was assessed using densitometry and is shown in Figure 6. A significantly higher level of skipping was observed for the two carotenoid lipids over a range of charge ratios compared to the same charge ratios of EPC lipid. The greatest level of exon 45 skipping for lipid C30-20 was observed at 68.3 ± 25.9% at a (+/−) charge ratio of 0.5:1 (p = 0.0493, paired t-test relative to the same charge ratio of EPC), and for lipid C20-20 was 29.7 ± 2.3%, at a (+/−) charge ratio of 0.25:1 (p = 0.0301, paired t-test, relative to the same charge ratio of EPC) with 250 nM leashed PMO (Figure 6). Charge ratios beyond 1:1 for both carotenoid formulations resulted in significant cell death (data not shown). At the (+/−) charge ratios tested, the two carotenoid lipids achieved greater exon 45 skipping in hSkMCs relative to the commercial lipid, EPC. A limitation of this study was the short differentiation time interval of twenty-four h used. As a result, the cells may not have been fully differentiated. This time point was chosen on the basis of previous experience from our lab with the commercial reagent, lipofectin (Invitrogen) [9,14]. Under identical experimental conditions in parallel experiments, up to 50% exon skipping was observed when using the commercial reagent, lipofectin (Invitrogen) at a ratio of 1:4 (data not shown). 

## 3. Experimental

### 3.1. Materials and Methods

Lipids EPC and cholesterol were obtained from Youjin Corporation (Seoul, Korea) and Avanti Polar Lipids (Alabaster, USA), respectively. PMO AO h45A30/1 (sequence available on request) was purchased from Gene Tools (Philomath, OR, USA), complementary leash h45A30/1L (sequence available on request) and RT-PCR primers from Eurofins MWG Operon (Ebersberg, Germany). Normal human skeletal muscle primary cells (hSkMCs) were purchased from TCS cellworks (Buckingham, UK), skeletal muscle cell growth and differentiation media plus supplements from PromoCell GmbH (Heidelberg, Germany), GeneScript RT-PCR system kit and 2× PCR Master Mix with cresol red from GeneSys Ltd. (Camberley, Surrey, UK). QIAshredder kit and RNeasy Mini kit were purchased from Qiagen Ltd. (Crawley, UK). Agarose, buffers and antibiotics were purchased from Invitrogen Ltd. (Paisley, UK). All solvents and chemical reagents were obtained from Sigma Aldrich (St. Louis, MO, USA) unless otherwise stated. Dichloromethane was obtained from Alfa Aesar (West Hill, MA, USA).

### 3.2. Synthesis of Cationic Carotenoid Lipids, C30-20 and C20-20

Lipids C30-20 and C20-20 were synthesized from commercial C30-carotenoid ester and C20-acid (retinoic acid). 2-Chloro-1,3,2-dioxaphospholane was reacted with dimethylamine, ethylene glycol, Br_2_ and 1-icosanol to bromoethylicosylhydroxyethyl phosphate, which was esterified with β-apo-8´carotenoic acid (C30 acid) and retinoic acid (C20 acid), followed by aminolysis to the lipids C30-20 and C20-20, respectively. The details will be published elsewhere.

### 3.3. Liposome and Lipoplex Formulation

#### 3.3.1. Ethanolic stock Solutions

Ethanolic stock solutions were made for each individual cationic lipid and co-lipid by dissolving a known amount of lipid in dichloromethane in a round-bottom flask. The dichloromethane solutions were placed on a rotary evaporator for two hours to obtain a film. The film was dissolved in a known amount of anhydrous alcohol, and once dissolved the alcohol stock was stored at −80 °C.

#### 3.3.2. Liposome Formulations

Hydrated liposomal (cationic lipid/co-lipid) formulations (namely, EPC/Chol, C30-20/Chol and C20-20/Chol, all at a cationic lipid/co-lipid molar ratio of 3:2) were generated from stock solutions from thin films by combining the required amounts of each alcohol solution of lipid and co-lipid, as determined by calculation of desired ratios, and removing the ethanol under reduced pressure. The thin films were then dissolved in a known amount of sterile water, followed by sonication to give a 2 mM final solution of hydrated stocks. These hydrated stock liposomal solutions were stored overnight at 4 °C. Before use, the hydrated stocks were warmed to 37 °C for 5 min in a water bath, then sonicated for 30 min.

#### 3.3.3. Preparation of Leashed PMO-AO

PMOs are unable to enter cells *in vitro* due to their lack of charge. Charge is introduced by annealing the PMOs to complementary phosphorothioate-capped oligodeoxynucleotide leashes. The complementary sequence of the PMO is 17 bases long, with tails at either end. The tails of the leash are always of the sequence ‘gattg’ (5’ to 3’) at the 5’ end of the PMO, and ‘gtgat’ (5’ to 3’) at the 3’ end of the PMO. Leash/PMO stocks were prepared at 100 µM by mixing 12.5 µL 10× PBS with 7.5 µL RNase-, DNase-free H_2_O, 25 µL leash (200 µM) and 5 µL PMO (1 mM) and annealed by gradual decrease in temperature from 94 °C in a thermal cycler, according to the method recommended by Gebski *et al.* [13]. Leashed PMOs were stored at 4 °C for a maximum of 6 weeks.

#### 3.3.4. Verification of Annealing PMO to Leash

Verification of annealing PMO to leash was established by running aliquots of leash alone and leashed PMO on a 3% agarose gel; an increase in size should be evident in leashed PMO relative to PMO alone, if PMO and leash have hybridized effectively.

#### 3.3.5. Lipid/PMO-AO Lipoplexes

Lipid/PMO-AO lipoplexes were formulated by adding equal volumes of liposome solution to PMO-AO at the desired charge ratio. The liposome particles were serially diluted to obtain varying cationic lipid/leash (N/P, or +/−) molar charge ratios at a given volume.

### 3.4. Bioassays

#### 3.4.1. Gel Retardation Assay

A gel retardation assay is a common technique, used in the context of this proposal to study the interaction between cationic lipids and AO. Briefly, the lipid/PMO-AO complexes, incubated in 20 mM HEPES, pH 5.5 were mixed with loading dye (bromophenol blue) and loaded onto the 3% agarose gel impregnated with ethidium bromide. The gel was then run at 105 V for 120 min in TBE buffer. The rate at which different molecules move through the gel was determined by their size and charge, and to a lesser extent, their shape.

#### 3.4.2. Transfection of normal human skeletal muscle primary cells (hSkMCs)

Cells were seeded in a 6-well culture plate at a density of 8 × 10^4^ cells/well and cultured till ~80% confluence. The growth medium was removed and replaced with 2 mL pre-warmed differentiation medium and cells incubated at 37 °C, 5% CO_2_ for 1 h. During this 1-hour incubation with differentiation medium, the lipid:leashed PMO:DMEM mixes were prepared and incubated for 30 min at RT. The differentiation medium was removed and wells were rinsed with 2 mL DMEM. A 1 mL aliquot of lipid:PMO:DMEM mix was added into each well and cells were incubated at 37 °C, 5% CO_2 _for 4 h. The lipid:PMO:DMEM mix was then replaced with 2 mL pre-warmed differentiation media. RNA was extracted after 24 h.

#### 3.4.3. RNA Extraction and Purification

RNA extraction and purification was performed using the QIAgen RNeasy mini kit and has previously been described [14]. In brief, cells were lysed with buffer RLT, and lysates were homogenized with a QIAshredder column. RNA was purified with a RNeasy mini column containing an silica-gel membrane, washed with RW1 and RPE buffers and eluted with RNase-free H_2_O and the concentration were measured using a ND-1000 Spectrophotometer (©Nano Drop). 

#### 3.4.4. RT-PCR

For assessment of exon 45 skipping 200 ng RNA was amplified by semi-nested RT-PCR. Primers used in the first round were forward h43f (5’-ACAACAAAGCTCAGGTCGGA-3’) and reverse h49r (5’ ATCTCTTCCACATCCGGTTG. RT-PCR was performed at 45 °C for 30 min, 92 °C for 5 min; then 20 cycles of 92 °C for 30 s, 60 °C for 30 s, 68 °C for 90 s; followed by 68 °C for 10 min in a reaction mix containing 300 nM of each primer, 200 mM dNTPs, 1.5 mM MgSO_4_ and 0.625 U GeneScript enzyme mix. A 2 mL aliquot of the first round product was further amplified in a second round using forward h44f (5’-CAGTGGCTAACAGAAGCTGAAC-3’) and reverse h48r (5’-CTTATGGGAGCACTTACAAGC-3’) primers. PCR was performed at 92 °C for 2 min, then 30 cycles of 92 °C for 30 s, 66 °C for 30 s and 68 °C for 90 s; followed by 68 °C for 10 min in a reaction mix containing 300 nM of each primer, 0.625 U Taq polymerase, 200 mM dNTPs and 1.5 nM MgCl_2_. For assessment of RNA quality between samples transfected with various lipid/PMO AO complexes, 100 ng of RNA was subjected to RT-PCR amplification using ribosomal 18s housekeeping primers. PCR amplification was performed at 92 °C for 2 min, then 30 cycles of 92 °C for 30 s, 60 °C for 30 s and 68 °C for 90 s, followed by 68 °C for 10 min in a reaction mix containing 100 nM of each primer, 0.625 U Taq Polymerase, 200 mM dNTPs and 1.5 nM MgCl_2_. The obtained products were separated by agarose gel (2.5%) electrophoresis against Hyper ladder V.

## 4. Conclusions

The novel cationic carotenoid lipids C30-20 and C20-20 were formulated into liposomes with the neutral co-lipid, cholesterol, as was the commercial cationic lipid, EPC. Each of these was subsequently formulated into lipoplexes containing leashed PMO capable of producing exon 45 skipping in hSkMCs; 29.7% exon skipping was achieved with C20-20/Cholesterol at a N/P (+/−) molar ratio of 0.25:1, and 68.3% with C30-20/Cholesterol, each at a N/P (+/−) molar charge ratio of 0.5:1. The C30 carotenoids performed significantly better at lower charge ratios as compared to the commercial cationic lipid, EPC. This study shows that carotenoid lipids have potential as delivery vectors for antisense oligonucleotides for exon skipping in Duchenne Muscular Dystrophy.
